# Long-term Effects of Desflurane and Sevoflurane on Mortality and Care Needs in Older Patients after Gastrointestinal Surgery: An Inverse Probability-weighted Analysis

**DOI:** 10.31662/jmaj.2025-0136

**Published:** 2025-10-03

**Authors:** Shinichiro Yoshida, Akira Babazono, Ning Liu, Reiko Yamao

**Affiliations:** 1 Department of Intensive Care Medicine, NHO, Kyushu Medical Center, Fukuoka, Japan; 2 Department of Health Care Administration and Management, Faculty of Medical Sciences, Kyushu University, Fukuoka, Japan; 3 Department of Preventive Medicine and Community Health, University of Occupational and Environmental Health, Kitakyushu, Japan; 4 Department of Public Health Nursing, School of Nursing, Faculty of Medicine, Fukuoka University, Fukuoka, Japan

**Keywords:** desflurane, sevoflurane, older adults, mortality, care-need level

## Abstract

**Introduction:**

Long-term mortality and activities of daily living (ADLs) outcomes in older patients who received desflurane anesthesia in real-world settings have not been evaluated. Therefore, we aimed to investigate whether the postoperative long-term care level of older patients who received desflurane was less impaired than that of patients who received sevoflurane, as well as the effects of desflurane on long-term mortality and long-term care-need level in older patients.

**Methods:**

We performed a retrospective, open-cohort study using medical and long-term care insurance (LTCI) claims data from Fukuoka Prefecture, Japan. Administrative medical claims data linked to LTCI claims data were analyzed. The study population included patients aged ≥75 years who underwent intra-abdominal surgery. The primary outcomes were 1-year mortality and deterioration in long-term care-need level after the desflurane and the sevoflurane anesthesia. An inverse probability weighting (IPW) analysis was performed to adjust for confounders. Generalized linear model analysis was performed to estimate the odds ratios (ORs) for the primary outcomes.

**Results:**

Among the 11,798 participants, 63.1% received sevoflurane and 36.9% received desflurane. The C-statistic of the propensity score for desflurane use was 0.701. In the generalized linear model analysis after IPW adjustment, desflurane did not cause deterioration in long-term care-need levels (OR, 0.931; 95% confidence interval [CI], 0.831-1.044) or reduced 1-year mortality (OR, 0.891; 95% CI, 0.782-1.016).

**Conclusions:**

Desflurane did not improve the long-term care-need level or 1-year mortality compared with sevoflurane in older patients. Therefore, we could not conclude whether desflurane improves long-term mortality or care levels.

## Introduction

Japan’s older population is rapidly increasing. Consequently, the number of surgical procedures performed in older patients is also increasing ^(1)^. Older patients are vulnerable and sensitive to surgical intervention, and postoperative outcomes, including mortality and physical function, are global concerns. In Japan, long-term care insurance (LTCI) is available to almost all residents aged ≥65 years. As of 2020, the Japanese government has certified approximately 6.8 million individuals based on the LTCI system for receiving any long-term care resources ^(2)^. The number of individuals requiring long-term care (LTC) has increased annually by approximately 2% ^(2)^. Among those certified by the LTCI system who are eligible for the use of supportive and care services with insurance benefits, 9.5%-23% of individuals who use any services throughout the year show a worsening of their care level by the next year ^(3)^. The incremental cost for individual service is approximately 281.3-578.0 USD per month (the value is calculated as follows: 1 USD = 131.498 JPY [the average rate in 2022]) if the care level worsens to the next level ^(4)^.

An enhanced recovery after surgery (ERAS) protocol has been developed for optimal perioperative care ^(5)^. The ERAS recommendations for standard anesthetic management address the depth of anesthesia in older populations ^(5)^. Because excessively deep anesthesia can cause postoperative confusion, the ERAS recommendations highlight the importance of rapid awakening. The finding that anesthetic choice may influence postoperative outcomes is important from an anesthesiologist’s perspective. However, studies examining the long-term effects of anesthetics on postoperative recovery in older patients are limited. Moreover, perioperative management of older patients by attending physicians and anesthetic management by anesthesiologists may affect postoperative outcomes. The challenge of studying postoperative outcomes is multifactorial adjustment because outcomes are influenced by several resources and processes. Therefore, researchers should consider using randomized controlled trials or multivariate analyses.

In Japan, sevoflurane is the most frequently used inhaled anesthetic, and it is also widely used worldwide. Desflurane has been used in Japan since 2011. Similar to sevoflurane, desflurane is widely administered. A notable characteristic of desflurane is rapid cognitive recovery. Moreover, previous studies have demonstrated that desflurane exhibits a distinct advantage over sevoflurane with respect to acceleration of postoperative recovery ^(6), (7), (8), (9), (10)^. However, the long-term effects of desflurane in older populations remain unclear. We hypothesized that desflurane anesthesia contributes toward maintaining the long-term care-need level and improving mortality compared with sevoflurane anesthesia in older patients undergoing major abdominal surgery. Therefore, we aimed to investigate the 1-year postoperative changes in the care needs of older patients who received desflurane and evaluate the long-term effects of desflurane.

## Materials and Methods

### Ethics approval

This study was approved by the Institutional Review Board of Kyushu University (Clinical Bioethics Committee of the Graduate School of Medical Sciences, Kyushu University [approval no. 2021-335]), which waived the requirement for informed consent for this noninterventional study because information from an anonymized data set was analyzed.

### Study design and data sources

We performed a retrospective open-cohort study using medical and LTCI claims data from Fukuoka Prefecture, Japan.

In 2021, the population of Fukuoka Prefecture was approximately 5,123,000, and approximately 14% were aged ≥75 years ^(11)^. The study database was provided by the Fukuoka Prefecture Wide-Area Association of Latter-Stage Elderly Healthcare (WALEH) for medical claims data and the Fukuoka Prefecture Wide-Area Association of LTCI (WALTC) for LTCI claims data. The WALEH covers approximately 95% of residents aged ≥75 years in Fukuoka Prefecture. The WALTC program provides universal coverage for almost all residents aged ≥65 years who require support or care for a specific disease or disability ^(12)^. The two databases contain the same identifier for each individual. Therefore, we can link both data sets and obtain information on the medical services and LTC for each individual.

The information in the WALEH database includes diagnosis, treatment, medication, prognosis, and incentives for special care. Special care includes pre- and post-anesthesia rounds by registered anesthesiologists, general anesthesia for patients with severe comorbidities, and intensive care. The information in the WALTC includes the amount of service used, length of stay at care facility, and patient’s LTC need level.

### Definition of LTC need level in the wide-area association of long-term care database

Under the LTCI system, care-need levels are classified into two subcategories of support levels and five subcategories of care-need levels according to the severity of comprehensive ability impairment in living, including physical and cognitive functions ^(12)^ (Supplementary Table 1).

### Study population

The surgical procedures selected for this study were gastric and colorectal malignant tumor surgeries, which are the most frequently performed malignant tumor surgeries in Japan. Surgical invasiveness is considered standard, and variations in surgical methods are unlikely to be substantial. We identified patients aged ≥75 years who underwent intra-abdominal surgery for gastrointestinal cancer, including gastrectomy, colectomy, and rectal resection, from April 2014 to March 2020 from the study database (Supplementary Table 2). Because we evaluated outcomes 1 year postoperatively, the study samples were identified as those for whom medical claims data and LTCI claims data could be collected for at least 1 year after surgery. Patients with a history of general anesthesia 1 year before or after surgery and those in whom multiple anesthetics were administered were excluded from the analysis. Patients who received total intravenous anesthesia were excluded from the final analysis.

### Study variables

We evaluated the care level and mortality rates 1 year postoperatively as the primary outcomes and compared the sevoflurane and desflurane anesthesia groups. These outcomes were the result of a multifactorial process; therefore, we adjusted for the relevant covariates.

The variables assumed to potentially influence outcomes were patient characteristics, such as sex, age, preoperative care level, and severity of comorbidities. In Japan, incentives are provided to manage intraoperative general anesthesia for patients with severe comorbidities and are approved by the Ministry of Health, Labour and Welfare (MHLW) (Supplementary Table 3). This information was obtained from the WALEH database and used to identify patients with severe comorbidities.

In addition, perioperative processes and management, including the operative site, procedure, combined anesthesia, intensive care unit (ICU) admission, and availability of pre- and post-anesthesia rounds by anesthesiologists, were considered to affect the outcomes. The WALEH database contains information on pre- and postoperative rounds by anesthesiologists, with different incentives depending on the positions in which the anesthesiologist completed the rounds. In brief, the highest-quality rounds are those performed by a registered anesthesiologist who is certified by the MHLW and meets specific requirements for anesthesia experience and duration of practice ^(13)^. Additional models of anesthetic rounds include rounds provided by a non-accredited anesthesiologist, such as a resident of anesthesiology supervised by a registered anesthesiologist. Moreover, anesthesiologist rounds may not be conducted as part of perioperative anesthesia care.

The number of hospital beds was also considered an institutional characteristic that may have affected the outcomes. Data on these factors were collected to provide results that were adjusted for covariates.

The Multipurpose Australian Comorbidity Scoring System (MACSS) was used to assess the severity of comorbidities and as a surrogate for physical status ^(14)^. In brief, the MACSS was calculated based on 17 major symptoms and 102 diseases and exhibited a better estimation of mortality than the Charlson Comorbidity Index ^(15)^. Toson et al. ^(15)^ calculated and validated the MACSS scores using the new International Classification of Diseases, Tenth Revision (ICD-10) codes. The study provided a table of correspondence between the ICD-10 codes used and disease status; 6,732 ICD-10 codes corresponded. In this study, we used the given correspondence table and matched it with the WALEH disease information to calculate the MACSS.

Surgical sites and procedures were obtained from the WALEH database by matching the selected operational codes (Supplementary Table 2). Information regarding the administered medications and blood products was obtained from the WALEH database by matching medication codes, including anesthetics. Transfusion of red blood cells within 1 week preoperatively and administration of chemotherapeutic agents within 3 months preoperatively were examined because we hypothesized that these treatments were more likely to negatively affect the physical status of the patients after surgery.

### Definition of LTC need level in this study

We obtained the LTC need level from the WALTC database every 6 months or 1 year if a patient was certified as an individual at the support and/or care-need level. If they used any services with WALTC benefits, their LTC need level was additionally recorded in the month in which they accessed the services. Therefore, we defined the LTC need level in the preoperative phase, which was recorded closest to the date of the operation and within a year before the operation. The LTC need level in the postoperative phase was defined as the closest to the date of 1 year from the operation and within 1 year postoperatively. If the LTC need level was not recorded 1 year preoperatively, we defined these individuals as patients without impairment or with no need for any support or care. If we could not obtain the LTC need level until 1 year postoperatively, we decided that the LTC need level did not change from the preoperative phase. We defined the deterioration of LTC need level as categorical worsening, that is, patients without LTC need level certification changed to support or care-need level. If patients certified as having a support need level changed to a care-need level, it was also considered deterioration. If the care level changed within a category (i.e., subcategorical change), we considered that deterioration was not observed.

### Statistical analyses

The 1-year postoperative survival rate in Japan was used to estimate the mortality rate in the sevoflurane group, which was assumed to be approximately 12%^(16), (17)^. In a previous study, the 1-year postoperative mortality difference between inhaled and intravenous anesthetics was reported to be −6.2% ^(18)^. We assumed that the difference between the desflurane and sevoflurane groups would not be as large as in the previous study but would be approximately half or more in the desflurane group. Assuming an α error of 5% and power of 80% to detect the assumed mortality difference, the desflurane group would be expected to be slightly smaller than the sevoflurane group, with a sample size ratio of approximately 1.5-fold. In this case, the total sample size for both groups was estimated to be at most 3,500 (desflurane group, 1,346; sevoflurane group, 2,154). Descriptive statistics were reported as numbers and percentages, medians, and interquartile ranges. Each variable was analyzed using the chi-square test for variables recorded as dichotomous measures, and the Kruskal-Wallis test was performed for continuous variables.

To match the variables and obtain less biased results, we performed an IPW analysis using the average treatment effect (ATE). From a practical perspective, cases where desflurane is indicated are compatible with sevoflurane. Consequently, it is reasonable to calculate the ATE. This method has the advantage of not reducing the sample size. Moreover, it can match variables between the two groups.

First, we calculated the propensity score with desflurane use as the dependent variable and sex, age, fiscal year, preoperative care level, lesion, laparoscopic surgery, concurrent epidural analgesia or peripheral nerve block, general anesthesia for severe comorbidities, availability of anesthesia rounds, ICU admission, preoperative red blood cell transfusion or chemotherapeutic agent administration, hospital bed size, and MACSS as the explanatory variables. After the determination of the propensity score for desflurane use, ATE was calculated based on the propensity score. We adopted a stabilized ATE to address extremely large weight values. Desflurane has been used in Japan since 2011. At the beginning of the study period, it is possible that desflurane created a bias in the patient population for which it was used. Consequently, this may result in the disproportionate weighting of patients during IPW implementation. Consequently, our policy was to calculate a stabilized ATE weight and use it to calculate the ATE. Finally, a generalized linear model analysis was performed to estimate the odds ratios (ORs) for the primary outcomes.

We confirmed the C-statistic of the propensity score for desflurane use and standardized the differences in each variable after weighting. This process required evaluation of the validity of the analysis. The C-statistic roughly indicates whether two patients receiving different anesthetics can be differentiated using explanatory variables. A low C-statistic (close to 0.5) indicates that the two groups are likely to be balanced, despite variations in the explanatory variables. In this case, if propensity score matching is used, some samples may be ineffectively lost. However, the IPW analysis can output results without the loss of samples after matching the variables.

All analyses were performed using the Stata/IC 14 software (StataCorp, College Station, TX, USA). All reported *p*-values were two-tailed, and the level of significance was set at p < 0.05.

## Results

**Figure 1.**
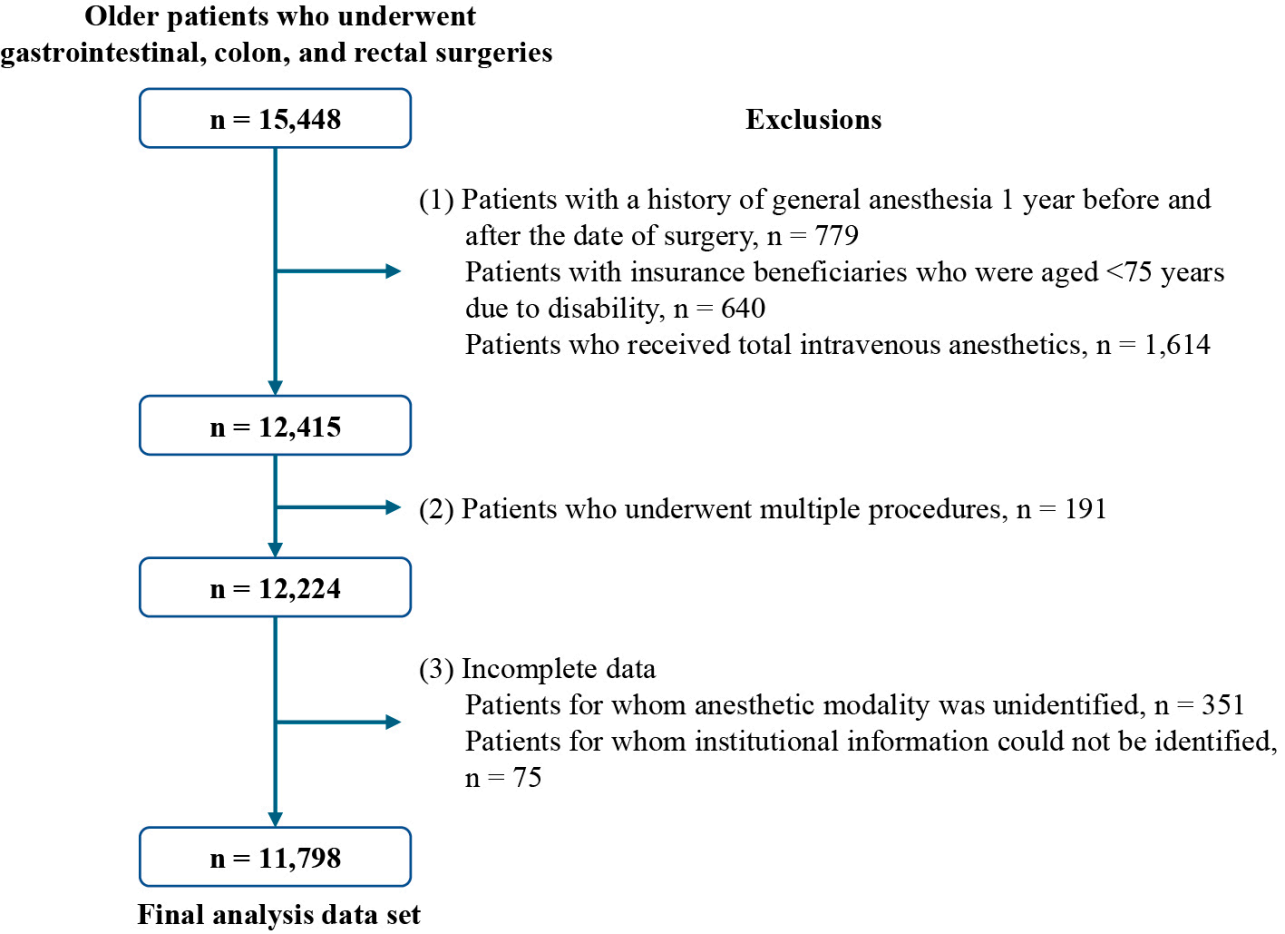
Flowchart of patient identification and selection. The following patients were excluded: (1) patients who had repeatedly received general anesthesia or primarily intravenous anesthetics because they could not be appropriately evaluated in terms of the effects of desflurane and patients who were older insurance beneficiaries with a disability (age, 65-75 years) because they might not be fairly evaluated; (2) patients who underwent multiple procedures during the same operation because they could have significant effects other than those of general anesthesia; and (3) patients with incomplete data.

We identified 14,478 patients who underwent selected surgical procedures during the study period, without repeat operations, 1 year before and 1 year after surgery. We excluded 2,680 patients because of the use of multiple anesthetics, intravenous anesthesia, discontinuation of insurance eligibility, and missing data. The final analyzed sample comprised 11,798 patients (Figure 1). Among these patients, 7,444 (63.1%) received sevoflurane anesthesia and 4,354 (36.9%) received desflurane anesthesia. Overall, 1,502 (12.7%) patients died within 1 year after surgery, whereas the care level of 2,032 (17.2%) patients deteriorated within 1 year postoperatively. Of those with a deterioration in care level, 367 (18.1%) died within 1 year postoperatively.

### Participant characteristics

Descriptive data showed that sex, age, lesion, postoperative ICU admission, and preoperative chemotherapy were not significantly different between the groups (Table 1). Notable variations (>10% difference between the groups) were observed in the fiscal year, incentives for pre- and post-anesthesia rounds, and number of hospital beds.

**Table 1.** Patient Distribution of Those Who Received Desflurane or Sevoflurane among the Study Variables.

Variables	Desflurane		Sevoflurane		p-Value	Standardized difference	
	n = 4,354		n = 7,444				
	n	%	n	%		Before weighted	After weighted
Sex, male	2,315	53.2	4,060	54.5	0.149	0.028	0.004
Age (median, IQR)	81	77-85	81	77-85	0.711		
75-79	1,786	41.0	3,045	40.9	0.893	-0.006	0.017
80-84	1,432	32.9	2,428	32.6			
≥85	1,136	26.1	1,971	26.5			
MACSS score (median, IQR)	4	3-6	4	3-7			
Mild (score, 0-3 points)	1,835	42.2	2,776	37.3	*0.000*	-0.106	-0.030
Moderate (score, 4-6 points)	1,496	34.4	2,658	35.7			
Severe (score, ≥7 points)	1,023	23.5	2,010	27.0			
Preoperative care support level							
N/A	3,363	77.2	5,642	75.8	*0.019*	-0.046	0.033
Support level	404	9.3	659	8.9			
Care level	587	13.5	1,143	15.4			
Preoperative care support level (detail)							
N/A	3,363	77.2	5,642	75.8	*0.029*		
Support level 1	234	5.4	327	4.4			
Support level 2	170	3.9	332	4.5			
Care level 1	225	5.2	444	6.0			
Care level 2	163	3.7	301	4.0			
Care level 3	106	2.4	204	2.7			
Care level 4	63	1.5	135	1.8			
Care level 5	30	0.7	59	0.8			
Fiscal year							
2014	391	9.0	1,433	19.3	*0.000*	0.264	0.034
2015	653	15.0	1,342	18.0			
2016	765	17.6	1,127	15.1			
2017	876	20.1	1,193	16.0			
2018	856	19.7	1,154	15.5			
2019	813	18.7	1,195	16.1			
Surgical site							
Gastric	1,314	30.2	2,193	29.5	0.701	-0.011	0.017
Colon	2,219	51.0	3,842	51.6			
Rectoanal	821	18.9	1,409	18.9			
Laparoscopic surgery	3,078	70.7	4,298	57.7	*0.000*	0.273	0.181
Local analgesia							
Epidural thoracic	3,180	73.0	5,507	74.0	*0.000*	0.118	0.008
Epidural other than thoracic	59	1.4	208	2.8			
Peripheral nerve block	272	6.3	188	2.5			
None	843	19.4	1,541	20.7			
Severe anesthesia (n, %)	950	21.8	1,482	19.9	*0.013*	0.047	0.002
Incentives for perioperative anesthetic management (n, %)							
None	1,012	23.2	3,134	42.1	*0.000*	0.390	0.018
By accredited anesthesiologist	2,466	56.6	3,351	45.0			
By non-accredited anesthesiologist	876	20.1	959	12.9			
Postoperative ICU admission (n, %)	794	18.2	1,347	18.1	0.848	0.004	0.018
Preoperative RBC transfusion (n, %)	272	6.3	571	7.7	*0.004*	-0.056	0.025
Preoperative chemotherapy (n, %)	17	0.4	39	0.5	0.309	-0.020	0.003
No. of hospital bed (n, %)							
≤99	189	4.3	467	6.3	*0.000*	0.429	0.027
100-199	162	3.7	1,395	18.7			
200-499	2,190	50.3	3,598	48.3			
≥500	1,813	41.6	1,984	26.7			

ICU: intensive care unit; IQR: interquartile range; RBC: red blood cell; MACSS: Multipurpose Australian Comorbidity Scoring System.

### IPW adjustment

The propensity score for desflurane use was calculated, and the C-statistic of the propensity score was 0.701 (95% confidence interval [CI], 0.692-0.711). Before IPW adjustment, the standardized mean difference in each anesthetic was −0.49 to 0.32; after IPW adjustment, the standardized mean difference decreased by −0.046 to 0.181. The absolute value of the standardized difference in the variables that showed notable differences was almost <0.1, indicating that these variables were well-balanced after IPW adjustment (Table 1).

### Primary outcomes

In the generalized linear model analysis after IPW adjustment, desflurane did not reduce the 1-year deterioration in the need for support or care (OR, 0.931; 95% CI, 0.831-1.044) or decrease 1-year mortality (OR, 0.891; 95% CI, 0.782-1.016) (Table 2).

**Table 2.** Results of Generalized Linear Model Analysis after ATE Weighting.

Outcomes	OR (95% CI)	SE	p Value
1-Year mortality of the desflurane group	0.891 (0.782-1.016)	0.059	0.084
1-Year support care level deterioration of the desflurane group	0.931 (0.831-1.044)	0.054	0.224

ATE: average treatment effect; CI: confidence interval; OR: odds ratio; SE: standard error.

## Discussion

In this study, we examined whether desflurane is superior to sevoflurane in terms of 1-year mortality and care level in older Japanese patients using administrative medical and LTC claims data. After balancing the patient characteristics, perioperative processes, and hospital profiles using the IPW approach, desflurane did not improve the 1-year mortality or the reduce care level. We used the propensity score for desflurane to calculate the ATE. Propensity matching was performed to obtain results which were consistent with those previously reported for ATE.

To the best of our knowledge, this is the first study to demonstrate the long-term outcomes of desflurane with respect to the need for support and care in older patients. We also clarified the occurrence of deterioration in LTC need level after volatile anesthesia in older patients. The strengths of our study are that we collected samples using real-world data, selected objective outcomes, and adjusted for several confounders. Although the causes of postoperative deterioration in LTC need level and postoperative death among older patients are multifactorial, we considered that the risk factors for the study outcome, except for perioperative management, were almost adjusted between the groups using the MACSS.

Several studies have reported the positive effects of desflurane, such as the recovery of cognitive function; however, they often have focused on short-term effects. We assumed that if some positive effects of desflurane are combined, outcomes may improve, especially in older populations, even if only short-term effects are observed because long-term outcomes are likely to be influenced by several factors, including anesthetic selection and the recovery process after anesthesia. Nevertheless, real-world data have shown that desflurane does not have advantages in terms of mortality and support or care needs.

One reason for these results is that desflurane may not fundamentally possess an advantage in terms of mortality and deterioration of care level. Several studies have reported no favorable effect on the length of hospital stay and stay in the post-anesthesia care unit ^(7), (19), (20)^. These studies have suggested that the favorable effects of desflurane may not persist over time. Therefore, desflurane might not be advantageous in terms of long-term outcomes, such as mortality and care level.

Second, other factors could have canceled out the favorable effects of desflurane. For example, attending physicians may not benefit from desflurane administration in a timely manner. Postoperative management may be provided according to a typical clinical pathway, regardless of the volatile anesthetic used. Moreover, the content of the clinical pathways, proportion of patients who adopted clinical pathways, and proportion of hospitals that established clinical pathways were unclear ^(21)^. The timing and intervention of perioperative rehabilitation remain controversial ^(21), (22), (23), (24)^, and providing optimal and timely interventions may lead to favorable outcomes.

Identifying patients with high-risk factors for poor outcomes may be important. Higher age, low cognitive function, dementia, and female sex are risk factors for reducing the need for supportive care, whereas male sex is a risk factor for mortality and hospitalization ^(25), (26), (27), (28)^. We may have to pay more attention to populations with these risks and proactively combine the benefits of desflurane with multimodal approaches.

This study has some limitations. First, the sample size was calculated based on the fact that the difference in mortality between the desflurane and sevoflurane groups was greater than 3%. However, if the observed difference in mortality was less than 2%, it is possible that the study sample size was insufficient to reach a definitive conclusion regarding the statistical significance of the observed difference in mortality. Second, we could not collect information regarding which function was impaired because the care level is a measure that indicates a comprehensive evaluation of care demand. If researchers can reveal the impaired function in detail, desflurane may contribute toward reducing the need for support or care. Third, the database used in this study did not contain information regarding cancer stage. Instead, we partially adjusted for cases with more advanced cancer stages by obtaining information on preoperative treatments, such as chemotherapy and blood transfusions. This study was based on the assumption that, except in rare cases, the surgical population did not exhibit poor short-term outcomes. Fourth, we were unable to collect laboratory data; examination results; American Society of Anesthesiologists Physical Status (ASA-PS) data; or information regarding the depth of anesthesia, amount of anesthetic consumption, and anesthesia duration. Although the MACSS was used alternatively for the preoperative physical status and adjusted for analysis, our results might change if the analysis is performed with adjustment for ASA-PS and other unmeasured confounders, such as anesthesia duration, intensity of perioperative rehabilitation, and postoperative complications. Lastly, the highest standardized difference was observed for laparoscopic surgery, and it is possible that laparoscopic surgery may have had an impact on desflurane outcomes. However, the standardized differences were minimal, and the overall impact on the outcomes was likely to be small.

We found no significant difference between the effects of sevoflurane and desflurane on long-term mortality. Although some studies have reported the effects of anesthetics on cancer progression, recurrence, and mortality ^(29), (30), (31)^, the debate regarding the effects of anesthetics on cancer remains at a nascent stage ^(32)^. If future studies provide evidence demonstrating the harmful effects of anesthetics on cancer outcomes, either or both anesthetics may not be used in cancer surgery ^(33)^.

In conclusion, compared with sevoflurane, desflurane did not improve 1-year mortality or reduce the need for support or care in older patients with gastrointestinal malignancies and surgery. These findings were based on an administrative database and adjusted for variables regarding patient characteristics, surgical procedures, and hospital profiles. We could not conclude whether desflurane improves long-term mortality or care level. Further investigation is required to confirm the robustness and accuracy of these results.

## Article Information

### Acknowledgments

The authors thank the Fukuoka Prefecture Wide-Area Association of Latter-Stage Elderly Health Insurance and the Fukuoka Prefecture Wide-Area Association of long-term care insurance for providing the medical and long-term care claims data.

### Author Contributions

Conceptualization: Shinichiro Yoshida and Akira Babazono. Data curation: Shinichiro Yoshida. Formal analysis: Shinichiro Yoshida, Akira Babazono, and Ning Liu. Writing - original draft: Shinichiro Yoshida. Writing - review and editing: Shinichiro Yoshida and Akira Babazono. Supervision: Ning Liu and Reiko Yamao. All authors interpreted the data, critically revised the manuscript for important intellectual content, and approved the final version of the manuscript.

### Conflicts of Interest

None

### Ethics Approval

This study was approved by the Institutional Review Board of Kyushu University (Clinical Bioethics Committee of the Graduate School of Medical Sciences, Kyushu University [approval no. 2021-335]), which waived the requirement for informed consent for this non-interventional study because information from an anonymized data set was analyzed.

## Supplement

Supplementary Material
