# Supplementary material for: Long-term Effects of Desflurane and Sevoflurane on Mortality and Care Needs in Older Patients after Gastrointestinal Surgery: An Inverse Probability-weighted Analysis
Source: JMA J. 2025 Oct 3;8(4):1331–8. doi: 10.31662/jmaj.2025-0136 (PMC12598310; doi:10.31662/jmaj.2025-0136)
Supplement: Supplementary Material [file 2433-3298-8-4-1331-s001.pdf]

Supplementary Table 1. National Standards and Characteristics of Support Care-need Level for Certification in Japan

<table>
<tr><th>Level</th><th>Estimated time of long-term care (min/day)*</th><th colspan="2">Description of status in each level</th></tr>
<tr><td>Support-need level 1</td><td>25–32</td><td colspan="2" rowspan="2">The basic abilities of daily living are mostly retained; however, some support is needed to prevent increased care needs.</td></tr>
<tr><td>Support-need level 2</td><td>32–50</td></tr>
<tr><td>Care-need level 1</td><td>32–50</td><td rowspan="3">Some care is needed because basic abilities of daily living are impaired.</td><td>Reduced abilities to perform instrumental activities of daily living compared with those at support need levels 1 and 2.</td></tr>
<tr><td>Care-need level 2</td><td>50–70</td><td>In addition to care-need level 1, partial support is needed for activities of daily living.</td></tr>
<tr><td>Care-need level 3</td><td>70–90</td><td>Compared with care-need level 2, almost complete care is needed because the abilities to perform activities of daily living and instrumental activities of daily living are substantially reduced.</td></tr>
</table>

Care-need level 4	90–110	In addition to care-need level 3, it is difficult to perform activities of daily living without care because movement capacity is further reduced.
Care-need level 5	≥110	It is almost impossible to perform daily living without care because movement capacity is more reduced than that at care-need level 4.

*The estimation of care time is calculated according to eight categories of care: eating, toileting, transferring, grooming/bathing, assistance with instrumental activities of daily living, behavioral problems, rehabilitation, and medical services

Supplementary Table 2. Procedure Names and Codes Used in This Study

Procedure	Procedure code for health insurance claims in Japan
Gastrectomy for malignancy	150168010
Laparoscopic gastrectomy for malignant neoplasm	150323510
Proximal gastrectomy for malignant neoplasm	150337310
Laparoscopic proximal gastrectomy for malignant neoplasm	150377910
Total gastrectomy for malignant neoplasm	150168110
Laparoscopic total gastrectomy for malignancy	150323710
Colectomy; total colectomy, subtotal colectomy, or colectomy for malignant neoplasm	150181910
Laparoscopic colectomy for malignant neoplasm	150324910
Anal surgery for malignant neoplasm	150190710
Anal surgery with rectal resection for malignant neoplasm	150264010
Rectal transection and resection, rectal transection	150187110
Rectal transection and resection, low anterior resection	150245410
Rectal transection and resection, ultra-low anterior resection	150297510
Rectal transection and resection, resection	150187210

Laparoscopic rectal transection and resection, rectal transection	150325210
Laparoscopic rectal transection and resection, low-anterior section	150337810
Laparoscopic rectal transection and resection, resection	150337910

Supplementary Table 3. Approved Conditions for Incentives for Difficult Anesthetic Management Due to Severe Comorbidities.

	Pre-anesthesia status
1	New York Heart Association functional class 3 or 4 heart failure
2	Canadian Cardiovascular Society functional class 3 or 4 angina pectoris
3	Myocardial infarction onset within 3 months
4	Aortic regurgitation, mitral regurgitation, and tricuspid regurgitation (≥grade 2)
5	Aortic stenosis (mean pressure gradient ≥50 mmHg), mitral stenosis (mean pressure gradient ≥10 mmHg)
6	Implanted pacemaker or defibrillator
7	Congenital heart disease (mean pulmonary artery pressure ≥25 mmHg)
8	Pulmonary arterial hypertension
9	Respiratory failure (partial pressure of oxygen [PaO_2] ≤60 mmHg or PaO_2/fraction of inspired oxygen ratio ≤300)
10	Ventilatory failure (forced expiratory volume in 1 s/forced vital capacity ratio ≤70% and actual vital capacity/estimated vital capacity ratio ≤70%)
11	Bronchial asthma (repetition of moderate attack despite treatment)
12	Diabetes mellitus (glycated hemoglobin ≥8.4% or fasting blood sugar ≥160 mg/dL or

	blood sugar at 2 h after meal ≥220 mg/dL)
13	Renal failure (serum creatinine ≥4.0 mg/dL)
14	Liver failure (Child–Pugh score B or more)
15	Anemia (hemoglobin ≤6.0 g/dL)
16	Coagulation disorder (prothrombin time-international normalized ratio ≥2.0)
17	Disseminated intravascular coagulation
18	Thrombocytopenia (platelet count <50,000/mcL)
19	Sepsis
20	Shock (systolic blood pressure <90 mmHg)
21	Complete spinal cord injury (thoracic level 5 or upper)
22	Mechanical support for cardiopulmonary insufficiency
23	Mechanical ventilation
24	Dialysis
25	Intra-aortic-balloon pumping
26	Body mass index ≥35 kg/m^2
